# Effects of Short-Term Tillage on Rhizosphere Soil Nitrogen Mineralization and Microbial Community Composition in Double-Cropping Rice Field

**DOI:** 10.4014/jmb.2401.01032

**Published:** 2024-06-18

**Authors:** Haiming Tang, Li Wen, Kaikai Cheng, Chao Li, Lihong Shi, Weiyan Li, Yong Guo, Xiaoping Xiao

**Affiliations:** Hunan Soil and Fertilizer Institute, Changsha 410125, P.R. China

**Keywords:** Rice, tillage, paddy field, N fertilization rate, soil microbial diversity

## Abstract

Soil extracellular enzyme plays a vital role in changing soil nitrogen (N) mineralization of rice field. However, the effects of soil extracellular enzyme activities (EEA) and microbial community composition response to N mineralization of rice field under short-term tillage treatment needed to be further explored. In this study, we investigated the impact of short-term (8-year) tillage practices on rhizosphere soil N transformation rate, soil enzyme activities, soil microbial community structure, and the N mineralization function gene abundances in double-cropping rice field in southern China. The experiment consisted of four tillage treatments: rotary tillage with crop straw input (RT), conventional tillage with crop straw input (CT), no-tillage with crop straw retention (NT), and rotary tillage with all crop straw removed as a control (RTO). The results indicated that the rhizosphere soil N transformation rate in paddy field under the NT and RTO treatments was significantly decreased compared to RT and CT treatments. In comparison to the NT and RTO treatments, soil protease, urease, *β*-glucosaminidase, and arginase activities were significantly improved by the CT treatment, as were abundances of soil *sub*, *npr*, and *chiA* with CT and RT treatments. Moreover, the overall diversity of soil bacterial communities in NT and RTO treatments was significantly lower than that in RT and CT treatments. Soil chitinolytic and bacterial ureolytic communities were also obviously changed under a combination of tillage and crop straw input practices.

## Introduction

Soil nitrogen is generally recognized as a major nutrient in agricultural soil and plays a vital role in changing crop growth. Recently, various problems in rice production have appeared due to excessive use of chemical N fertilizer. These problems include soil degradation, non-point source pollution, increase of nitrate leaching, and greenhouse gas emissions [1]. Effective practices for resolving these issues are efficient use of inorganic N fertilizer and controlled input level of N fertilizer in paddy field. Previous findings showed a close correlation between tillage practices, soil N mineralization, and microbial community structure [2]. These were seen as positive practices for promoting soil quality and improving the ecological environment in agricultural soil under tillage and returning-crop-straw conditions [3].

Soil N mineralization plays a significant role in controlling conversion of N in rice field, and is obviously influenced by planting system, tillage, and returning straw. It is usually accepted that soil N mineralization in rice field changes with tillage practices. Previous studies found that both soil aerobic and anaerobic N mineralization rates in rice field with tillage managements were increased [4]. Soil potential for mineralizable N and maximum nitrification rate were enhanced under different tillage practice conditions [5]. However, another study showed that soil N mineralization with inversion tillage treatment was lower than that of no-tillage treatment [6]. Mahal *et al*. [7] found that soil potentially mineralizable N with no-tillage treatment was obviously improved, compared to moldboard plowing. Li *et al*. [8] found a positive correlation between soil N mineralization rate and soil enzyme activities, but there was a negative correlation between soil properties and soil aerobic N mineralization rate in rice field. These results were closely related to soil physicochemical and microbiological characteristics [9, 10].

Soil extracellular enzyme activities (*e.g.*, soil *β*-glucosidase, arylamidase, *β*-glucosaminidase, L-glutaminase, and urease) obviously play a role in improving soil physicochemical and microbiological characteristics, and are generally considered an effective index of soil nutrient cycling and soil environment, which are closely related to different tillage practices. Meanwhile, soil enzyme activities were obviously improved under a higher level of soil nutrient conditions, and there was a close correlation between soil N mineralization and its enzyme activities [11]. Soil *β*-glucosaminidase and arylamidase activities play a vital role in changing the formation of easily hydrolyzable soil N fraction [12]. Soil enzymes activities (*e.g.*, soil urease, catalase, invertase, β‐glucosidase, acid phosphatase, and cellulase) under conservation tillage (CT) and crop straw mulching practices were improved by comparison with no-tillage (NT) treatment [13]. Some results showed that soil β‐glucosidase and urease activities with CT treatment were significantly higher than that of NT treatment [14]. Other results showed that soil urease, β‐glucosidase, and acid phosphatase activities with NT treatment were obviously higher, while *Proteobacteria* and *Verrucomicrobia* with NT treatment were also more abundant, and *Bacteroidetes* with CT treatment were significantly increased [15]. Soil *β*-glucosaminidase activity was reduced, but soil urease activity was enhanced with strip tillage (ST), compared with CT treatment [16]. There is a positive correlation between soil mineralizable N and soil extracellular enzyme activities (soil urease, amidohydrolase and L-asparaginase activities) [17]. Moreover, there is a positive correlation between soil mineralizable N and soil urease activities, and a negative relationship with soil *β*-glucosidase and dehydrogenase activities [16]. However, there is still a need to further investigate specific enzyme activity related to soil N mineralization, as well as the correlation between soil N mineralization, soil microorganism community structure, and soil enzymes activities.

The area of land used for rice production in the Asian region accounts for the highest proportion in the world [18]. Tillage and crop straw input are positive practices that have been used to enhance soil fertility and soil environment in rice field. Our previous results demonstrated that there was an obvious difference in the soil chemical and physical characteristics of paddy fields under different tillage management, including soil organic carbon (SOC), N content, bulk density, and pH [19, 20]. Our results were consistent with previous findings [6], which showed that soil fertility and soil N mineralization in rice field with NT treatment were obviously improved. However, the response to different tillage practices of rhizosphere soil N transformation rate, soil N mineralization functional gene abundances, soil microbial community structure (chitinolytic (*chiA*) and bacterial ureolytic (*ureC*)), and soil enzyme activities in the double-cropping rice field need to be further explored. To accomplish this goal, a short-term (8-year) field tillage experiment was established under the double-cropping rice system. Therefore, the objective of the present experiment was: (1) to explore the characteristics of rhizosphere soil N transformation rate and enzyme activities in rice field under various tillage conditions; (2) and, to investigate the rhizosphere soil microbial community structure and function in rice field under various tillage management practices.

## Materials and Methods

### Experimental Field Conditions

The field experiment was located in the main double-cropping rice production area in Ningxiang City (28°07'N, 112°18' E), Hunan Province, China. Conditions regarding monthly mean temperature, annual mean precipitation, evapotranspiration in the field experiment region, soil physicochemical characteristics at the arable layer (0-20 cm) in the rice field before starting field experiment, and planting system followed those previously reported by Tang *et al*. [19].

### Experimental Design

The field experiment was begun in November 2015, and included four tillage treatments: rotary tillage with crop straw input (RT), conventional tillage with crop straw input (CT), no-tillage with crop straw retention (NT), and rotary tillage with all crop straw removed as a control (RTO). Each plot area measured 56.0 m2, and was laid out in a randomized complete block design with triple repetition. Tillage management practices, amount of crop straw returning to rice field and inorganic fertilizer, rice varieties, date of rice transplanting and harvesting, and irrigation and weed control in paddy field followed those reported by Tang *et al*. [19].

### Soil Sample Collection

Rhizosphere soil samples were collected at the maturity stage of late rice in October 2022. The information regarding method and number of rhizosphere soil samples followed that reported by Tang *et al*. (2021) [4]. All soil samples were divided into two parts after removing the foreign matter (*e.g.*, stones, root debris, etc.). One part of the soil sample was stored at 4°C to investigate soil nitrogen transformation rate and soil enzyme activities, and the other parts were kept at −20°C for extracting soil DNA.

### Laboratory Analysis of Soil

**Soil N Transformation Rate**

The gross N transformation rate of soil samples was measured by using a ^15^N pool dilution. Soil ammonium (NH_4_^+^) or nitrogen dioxide (NO_2_^-^) plus nitrate ion (NO_3_^-^) contents were investigated with a flow injection analyzer, and the measurements were conducted as previously reported [21]. Soil net mineralization and nitrification were investigated after a 21-day incubation period. Headspace carbon dioxide (CO_2_) was determined at days 3, 7, 14, and 21 using a gas chromatograph to measure soil respiration rate.

### Soil Extracellular Enzyme Activities

Soil extracellular enzyme activities (EEAs) were measured after day 7 pre-incubation, using methods previously described [22], and covering soil urease (EC 3.5.1.5), protease (EC 3.4.21), *β*-glucosaminidase (EC 3.21.30) and arginase (EC 3.5.3.1). Briefly, substrate was added and then the soil enzyme activity reaction was stopped in the control after completion of the cultivation experiment. Soil EEAs were expressed according to the quality of non-moisture soil.

### Soil DNA Extraction and Real-Time Quantitative PCR

Soil sample DNA extraction and quantification were conducted according to the manufacturer’s protocols. PCR detection of enzyme-encoding genes related to soil nitrogen mineralization was conducted by using a CFX Connect Real-Time PCR measurement system (Bio-Rad, USA) and SsoAdvanced SYBR Green Supermix (Bio-Rad). Meanwhile, abundances of metalloprotease-encoding genes (*npr*), subtilisin (*sub*), urease (*ureC*), and chitinase (*chiA*) in the soil samples were measured. Primer, amplification condition, efficiency, and calibration standard of bacterial isolates (*npr*, *sub*, *ureC* and *chiA*) were conducted as previously described [23].

### Soil Metagenome Processing and Gene-Targeted Assembly

Soil DNA was sequenced based on the Illumina HiSeq 2500 platform using a paired-end configuration of 2 × 150 bp. Quality filtered metagenomes were checked and used for gene-targeted assembly. Nitrogen mineralization-related genes (*npr*, *sub*, *ureC* and *chiA*) were recovered based on targeted assembly. For each gene of interest, seed sequence, hidden Markov model (HMM), and nucleotide and protein reference sequences were downloaded from FunGene [24]. Sequences were clustered using Quantitative Insights into Microbiological Ecology (QIIME, version 1.7.0) with 95% amino acid similarity. Representative sequences from each cluster were searched using BLAST to compare the reference gene database and non- redundant database (*nr*) from the National Center for Biotechnology Information (NCBI). Overall, these representative sequences showed a similarity of over 49% with the highest hit rate of the reference gene database and an E-value of over 1.5 E_46.

### Illumina Sequencing and Data Analysis for *ureC* and *chiA*

Sequencing of *chiA* and *ureC* amplicon libraries were completed for soil sampled with different tillage treatments in October 2022. The same *ureC* and *chiA* primers mentioned above were used for high-throughput sequencing. We added linkers to primers for *ureC* and *chiA* genes and labeled them to separate various soil samples [25]. The same quantity of soil DNA was used for *ureC* and *chiA* amplification, and the PCR products were further purified through size selection. The collected purified products were sequenced based on the Illumina MiSeq platform (Illumina, Inc., USA) by using V3 chemistry (2 × 300 bp paired-end sequencing).

High-quality *ureC* and *chiA* sequences were extracted from merged reads of all soil samples by using the Ribosomal Database Project (RDP) SeqFilters with a cutoff Q score of 25. Chimeric sequences were deleted using UCHIME, and the *ureC* and *chiA* nucleotide reference databases were downloaded from FunGene [24]. The remaining quality screened protein sequences of all soil samples were aligned by using HMMER3 according to *ureC* and *chiA* hidden Markov models. Operational taxonomic units (OTUs) were clustered with 95% amino acid similarity by using the RDP clustering tool. A phylogenetic tree was constructed from representative sequences by using FastTree with default parameters. The threshold for bootstrap values was 92% and three replicates were used [26]. OTU tables and classification files were organized for analysis of diversity by using the R package phyloseq [27].

### Illumina Sequencing of 16S rRNA

The V4 variable region of the 16S gene was amplified with 515F and 816R primers for soil bacterial community. The 16S amplicon sequencing was performed based on an Illumina MiSeq instrument (Illumina Inc.). The Illumina raw reads were processed by using a custom pipeline. The quality filtering, taxonomies assigned, and data files organized were conducted as previously described [23, 28]. Illumina sequence data on *ureC* and *chiA* were saved in the NCBI under the BioProject login number PRJNA597781. The Illumina sequence data on 16S rRNA can be obtained under NCBI project ID 1061777.

### Statistical Analysis

All survey items with different tillage practices were represented by mean and standard deviation. The data for each measured index with all tillage practices were compared by using one-way analysis of variance (ANOVA) according to standard procedures at a probability level of 5%. In addition, the distance matrices were visualized and assessed with nonmetric multidimensional scaling (NMDS) and two-way permutational multivariate analysis of variance (PERMANOVA) by using the R package vegan. Impacts of various tillage practices on functional gene abundances and prokaryotic community alpha diversity were analyzed with the two-way ANOVA method. All data for every item of measurement in the present article were analyzed by using the SAS 9.3 software package [29].

## Results

### Soil Nitrogen Transformation Rate and Enzyme Activities

Our results showed that the gross nitrogen (N) mineralization rate (GMR) with CT treatment was significantly (*p* < 0.05) increased, compared to NT, RT, and RTO treatments. Soil GMR with CT treatment was enhanced by 16.77%, compared to NT treatment. Meanwhile, compared to NT and RTO treatments, soil gross ammonium consumption (GACR), net mineralization (NMR), gross nitrification (GNR), gross nitrate consumption (GNCR) and net nitrification rates (NNR) with CT and RT treatments were significantly (*p*<0.05) increased. There were also significant differences (*p* < 0.05) in the rates between CT, RT, and NT treatments. Compared to NT treatment, soil GACR, GNR, GNCR, NMR and NNR with CT treatment were enhanced by 17.49, 90.70, 78.13, 93.33 and 77.14%, respectively. Soil respiration rate (RR) with CT and RT treatments was significantly (*p*<0.05) higher than that of NT and RTO treatments. Soil RR with RT and CT treatments was respectively enhanced by 10.73 and 13.09% compared to NT treatment.[Table T1]

**Fig. 1:**
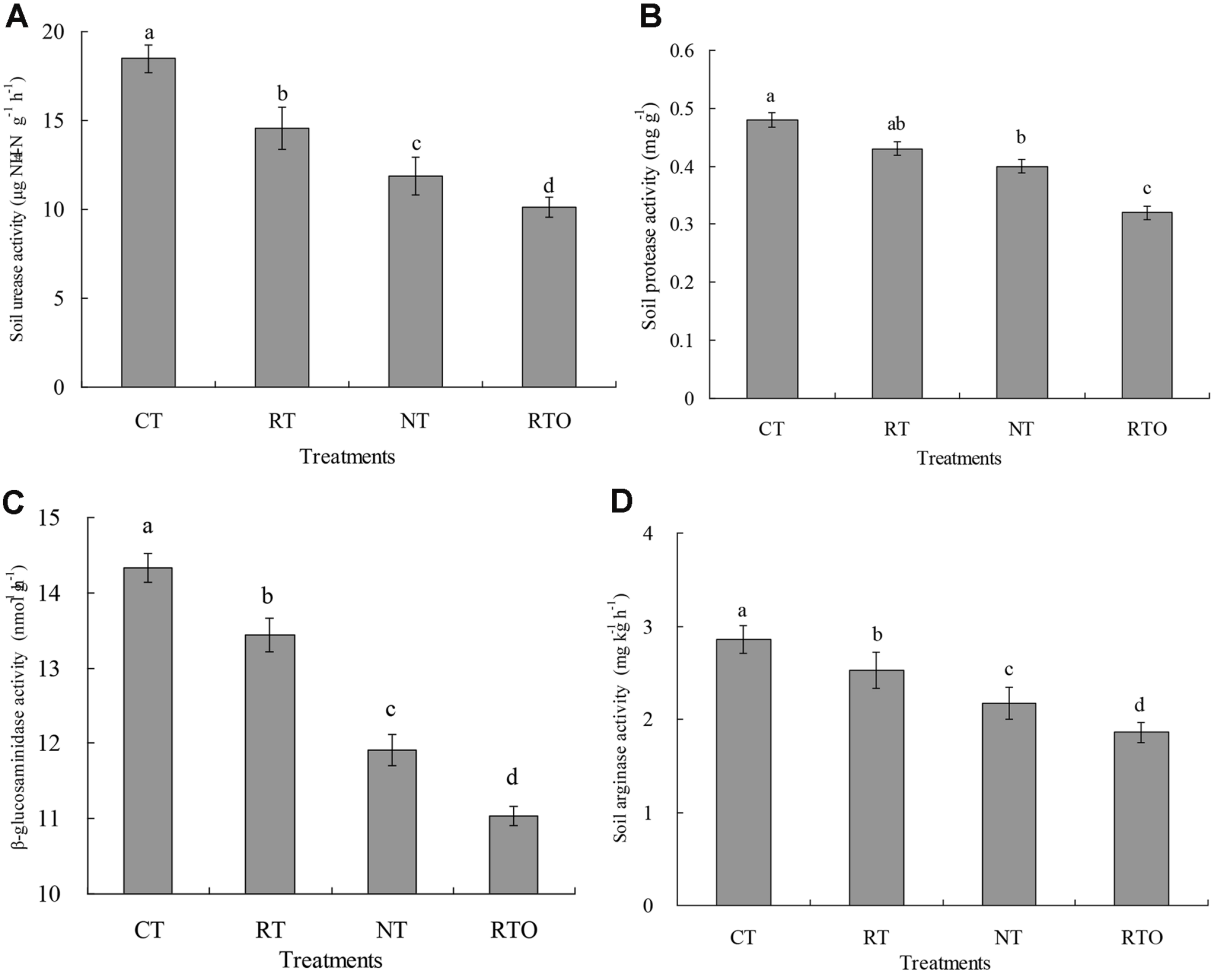
Effects of different short-term tillage treatments on rhizosphere soil EEA in double-cropping rice field. RT: rotary tillage with crop straw input; CT: conventional tillage with crop straw input; NT: no-tillage with crop straw returning; RTO: rotary tillage with all crop straws removed as a control. (**A**) was soil urease; (**B**) was soil protease; (**C**) was soil *β*-glucosaminidase; (**D**) was soil arginase. Different lowercase letters indicated significant differences at *p* < 0.05 among different tillage treatments. Values were presented as mean ± SE.

The impacts of tillage managements on rhizosphere soil EEA (soil urease, arginase, *β*-glucosaminidase and protease) in paddy field were shown in Fig. 1. These results indicated that soil urease activity in paddy field with CT and RT treatments was significantly (*p* < 0.05) higher than that of RTO and NT treatments. There were also significant differences (*p* < 0.05) in soil urease activity between CT and RT treatments. Compared to NT treatment, soil urease activity with RT and CT treatments was enhanced by 22.75 and 55.69% (Fig. 1A). This result indicated that soil protease activity with NT and RTO treatments was significantly (*p* < 0.05) lower than that of CT treatment. Compared with NT and RTO treatments, soil protease activity with CT treatment was enhanced by 20.00 and 45.45% (Fig. 1B). Our result showed that soil *β*-glucosaminidase and arginase activities with RT and CT treatments were significantly (*p* < 0.05) improved compared to RTO and NT treatments (Fig. 1C and 1D). Compared to NT treatment, soil *β*-glucosaminidase and arginase activities with CT treatment were enhanced by 20.32 and 31.80% (Fig. 1C and 1D).

### Gene Abundances in Soil N Mineralization

The various rhizosphere soil abundances of *sub*, *npr*, *chiA* and *ureC* in paddy field with all tillage treatments were indicated in Table 2. This result proved that the abundance range of *sub* with all tillage practices was 2.41 to 3.85 × 107 copies/g. Meanwhile, compared to NT and RTO treatments, the abundance of *sub* with RT and CT treatments was significantly (*p* < 0.05) improved, and the abundance of *sub* with RT and CT treatments was enhanced by 29.72 and 34.62%, respectively. The abundance ranges of *npr* and *chiA* with all tillage treatments were 2.23 to 2.91 × 10^5^ and 2.24 to 3.05 × 10^8^ copies/g, respectively. Meanwhile, the abundances of *npr* and *chiA* with RT and CT treatments were significantly higher (*p* < 0.05) than that of RTO and NT treatments. Compared to NT treatment, the abundances of *npr* and *chiA* with CT treatment were enhanced by 18.29 and 18.22%, respectively. Our result proved that the abundance range of *ureC* with all tillage treatments was 7.47 to 10.25 × 10^7^ copies/g. The abundance of *ureC* with CT treatment was significantly (*p* < 0.05) increased compared to RT, NT, and RTO treatments. Compared to RT and NT treatments, the abundance of *ureC* with CT treatment was enhanced by 5.89 and 14.78%. The average abundances of *sub*, *npr*, *chiA* and *ureC* with all tillage treatments were 3.21 × 10^7^, 2.60 × 10^5^, 2.71 × 10^8^, and 9.08 × 10^7^ copies/g, respectively (Table 2).

### Bacterial Community Composition

**Fig. 2:**
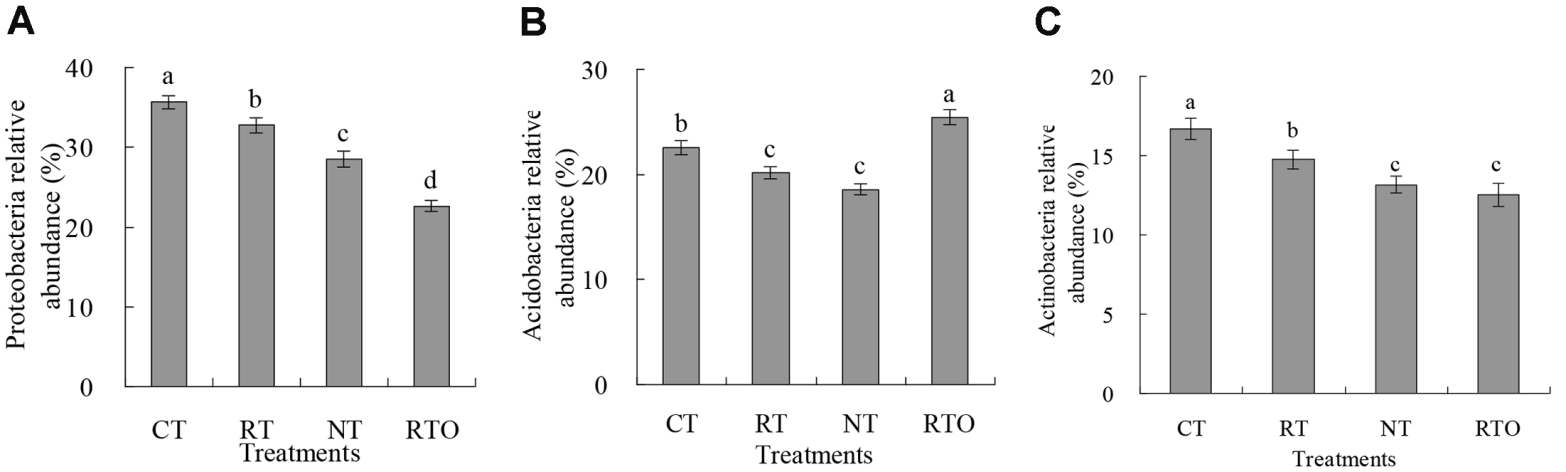
Effects of different short-term tillage treatments on rhizosphere soil abundances of *Acidobacteria*, *Actinobacteria*, and *Proteobacteria* in double-cropping rice field. (**A**) was *Proteobacteria*; (**B**) was *Acidobacteria*; (**C**) was *Actinobacteria*. Different lowercase letters indicated significant differences at *p*<0.05 among different tillage treatments. Values were presented as mean ± SE.

The impacts of tillage treatments on rhizosphere soil abundances of *Acidobacteria*, *Actinobacteria*, and *Proteobacteria* in rice field were indicated in Fig. 2. This result proved that *Acidobacteria*, *Actinobacteria*, *Gemmatimonadetes*, *Bacteroidetes*, and *Proteobacteria* were the most abundant of the five phyla, which accounted for over 82.5% of the abundance of soil bacterial communities. Meanwhile, our result indicated that abundances of *Actinobacteria*, *Acidobacteria*, and *Proteobacteria* were significantly changed with tillage treatments (Fig. 2). Abundances of *Actinobacteria* and *Proteobacteria* with RT and CT treatments were enhanced, and the abundance of *Acidobacteria* with NT, RT, and CT treatments were decreased. Abundances of *Actinobacteria* and *Proteobacteria* with RTO and NT treatments were significantly (*p* < 0.05) lower than that of CT and RT treatments. However, compared to NT, RT, and CT treatments, the abundance of *Acidobacteria* with RTO treatment was significantly (*p* < 0.05) increased.

**Fig. 3:**
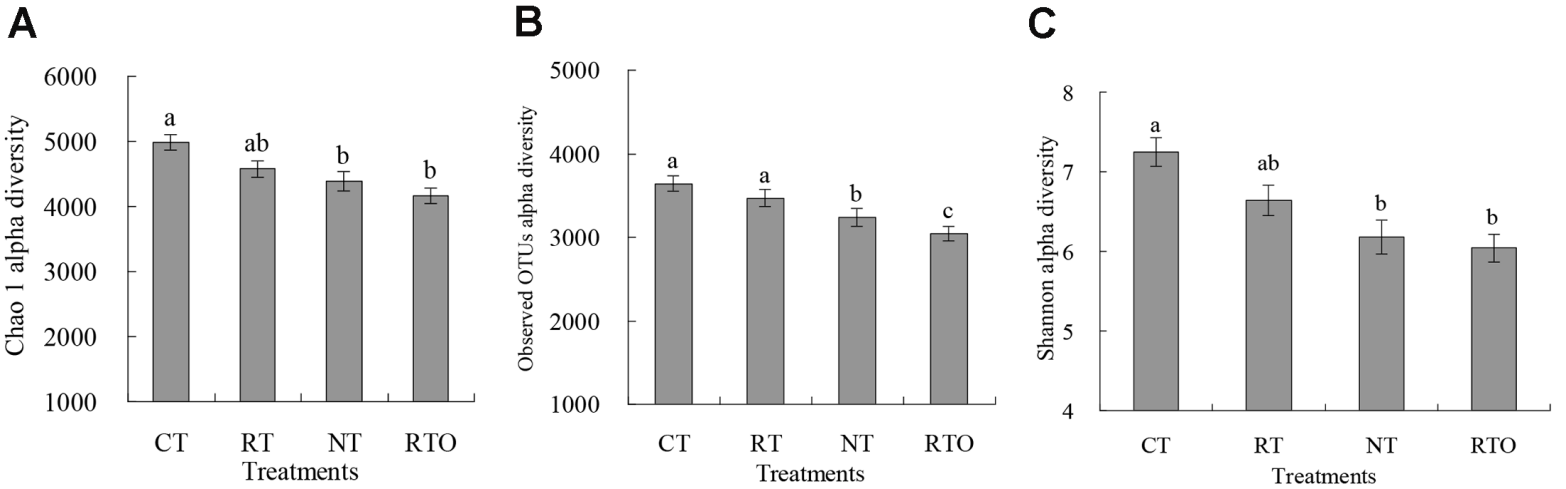
Effects of different short-term tillage treatments on alpha diversity of rhizosphere soil bacterial community in double-cropping rice field. (**A**) was Chao 1; (**B**) was observed OTUs; (**C**) was Shannon. Different lowercase letters indicated significant differences at *p* < 0.05 among different tillage treatments. Values were presented as mean ± SE.

This result proved that the alpha diversity of the rhizosphere soil bacterial community was obviously influenced with different tillage practices (Fig. 3). Meanwhile, our result indicated that Chao 1 and Shannon diversity with CT treatment were obviously (*p* < 0.05) increased compared to RTO treatment. However, there were no obvious differences (*p* > 0.05) in Chao 1 and Shannon diversity between RTO and NT treatments. Our result demonstrated that the observed OTU diversities with RT and CT treatments were significantly (*p* < 0.05) higher than that of RTO and NT treatments.

**Fig. 4:**
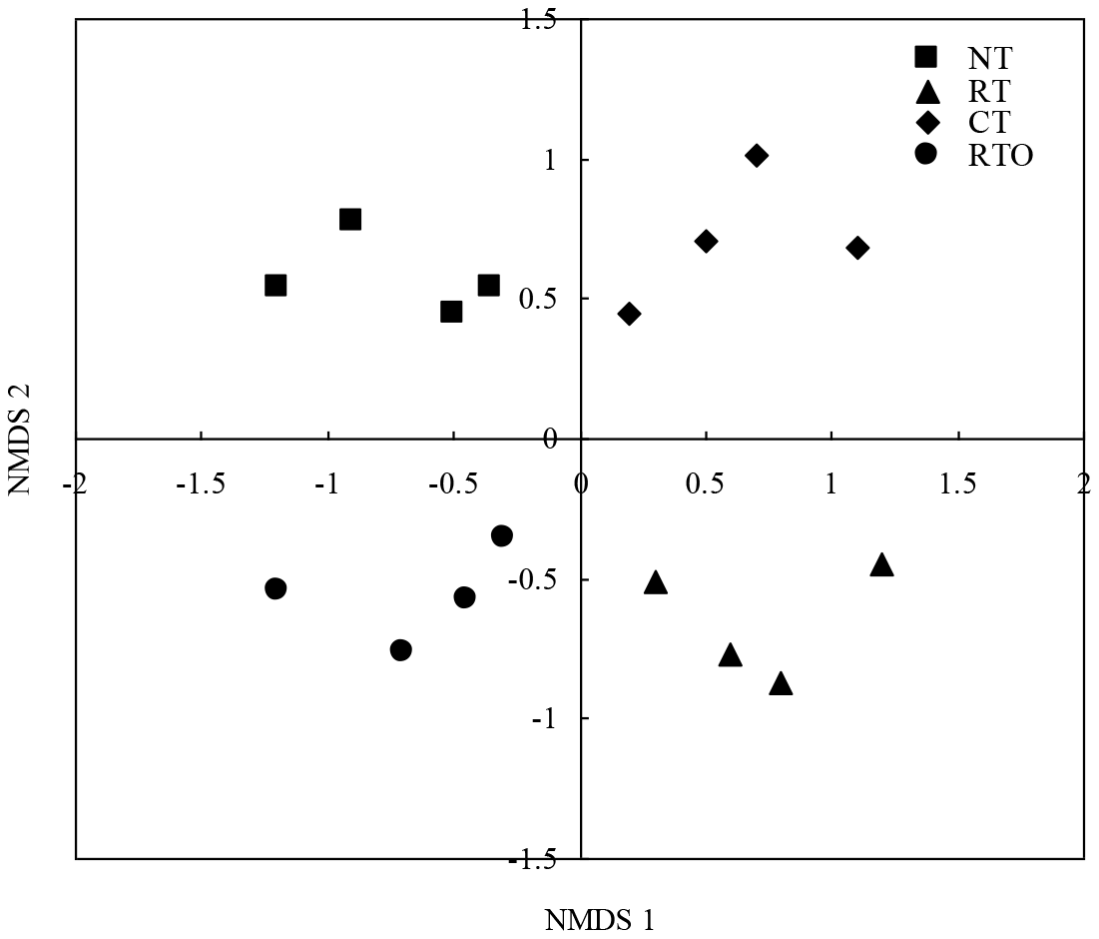
Nonmetric multidimensional scaling (NMDS) ordination (stress=0.1) of the weighted UniFrac distance for rhizosphere soil bacterial community with different short-term tillage treatments.

The differences in weighted UniFrac distance for rhizosphere soil bacterial community structure with different tillage practices were analyzed. The results proved that soil bacterial community structures were obviously distinct according to RTO, RT, CT, and NT treatments (Fig. 4). Meanwhile, by the PERMANOVA method (*p* = 0.004), our results further demonstrated that the soil bacterial community structure was significantly altered by various tillage treatments.

**Fig. 5:**
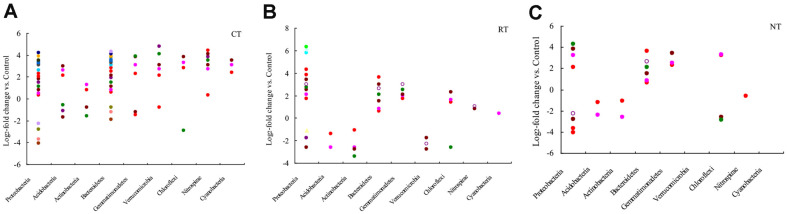
Log2-fold change in relative abundance of OTUs compared with those of the RTO treatment in rhizosphere soil. Each circle represents a single OTU with an adjusted *p*-value of <0.1.

Based on various log2-fold OTU abundances, the impacts of tillage management on rhizosphere soil OTUs in paddy field were shown in Fig. 5. Most of these reactive OTUs mainly come from *Bacteroidetes* and *Proteobacteria*, which were increased by the combined application of tillage and crop straw input practices. This result showed that there were 40, 55, and 72 responsive OTUs in rhizosphere soil treated with NT, RT, and CT treatments, respectively. Soil treated with RT and CT treatments shared 50% of their reactive OTUs, while a lower 7% of reactive OTUs were shared with RTO treatment.

### Ureolytic Community Composition

**Fig. 6:**
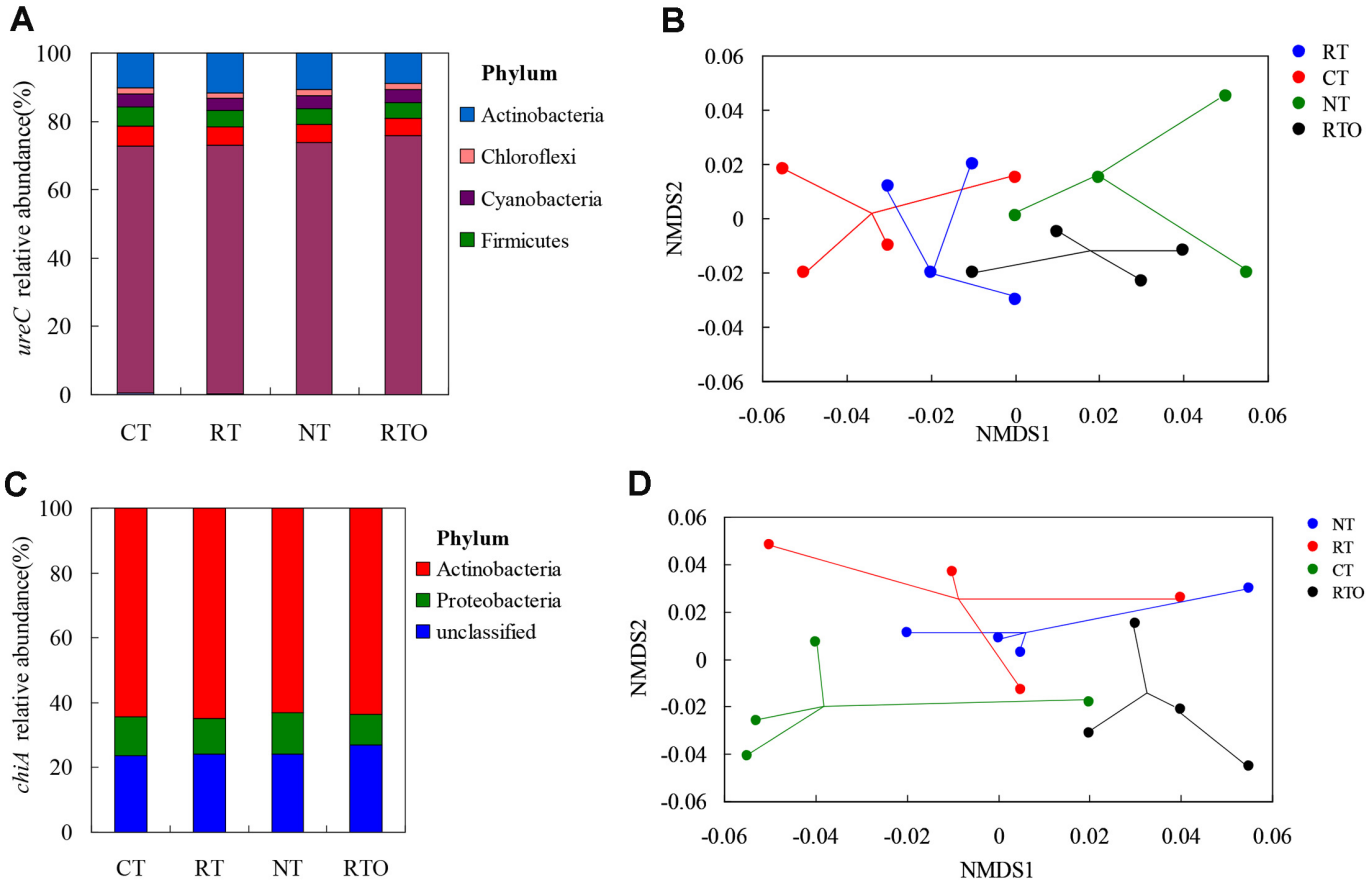
Effects of different short-term tillage treatments on relative abundance of rhizosphere soil bacterial community in double-cropping rice field. (**A**) Relative abundances of the dominant phyla (>1%) for bacterial *ureC*. (**B**) Nonmetric multidimensional scaling (**NMDS**) ordination (stress=0.09) of the weighted UniFrac distance for bacterial *ureC* under four tillage treatments. (**C**) Relative abundances of the dominant phyla (>1%) for bacterial *chiA*. (**D**) Nonmetric multidimensional scaling (**NMDS**) ordination (stress=0.05) of the weighted UniFrac distance for bacterial *chiA* under four tillage treatments.

Our results showed that 11 distinct prokaryotic phyla were detected according to the nearest matching reference taxonomy (Fig. 6A). Most sequences belong to *Proteobacteria* (76%), *Actinobacteria* (11%), and *Nitrospirae* (5%). The lowest relative abundance was *Thaumarchaeota* (0.05%) among these phyla with all tillage treatments. There were obvious differences (*p* < 0.05) in rhizosphere soil abundance of phyla among different tillage treatments. This result indicated that soil ureolytic community compositions obviously varied with tillage practices (*p* = 0.005), based on weighted UniFrac distance matrices (Fig. 6B). Meanwhile, our result further proved that RTO treatment showed obvious differences from compositions under CT (*p* = 0.025) and RT (*p* = 0.032) treatments.

**Fig. 7:**
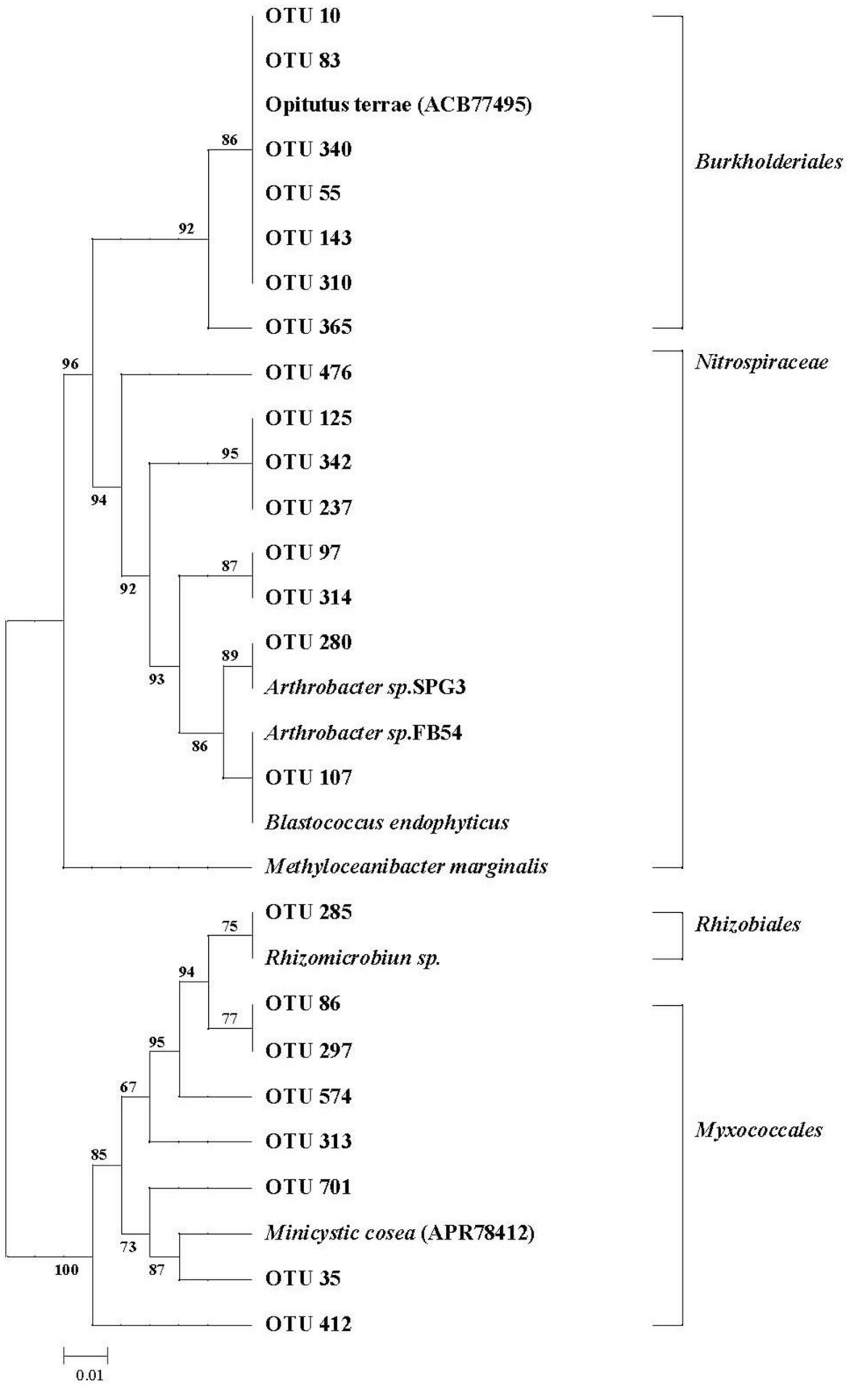
Phylogenetic tree of the top 500 most abundant partial *ureC* OTUs. Different branches indicated different taxa of *ureC*. OTUs: Operational taxonomic units.

This result indicated that *ureC* OTUs were mainly distributed among *Proteobacteria* with family members coming from *Burkholderiales*, *Myxococcales*, and *Rhizobiales*. OTU 285 was not grouped with other taxa of *Proteobacteria*, but was closely related to *Rhizobiales*. OTU 412 and OTU 125 belonged to the families *Myxococcales* and *Nitrospiraceae*, respectively. Among the top 500 *ureC* OTUs, *ureC* OTU abundances were obviously varied under different tillage conditions (Fig. 7).

### Chitinolytic Community Composition

Our result indicated that OTUs mainly belonged to *Proteobacteria* and *Actinobacteria* according to the closest matching reference taxonomy (Fig. 6C). The abundance of *Proteobacteria* was highest, but the abundance of *Actinobacteria* was lowest with NT treatment. This result showed that abundance of *Proteobacteria* with CT and NT treatments was significantly (*p* < 0.05) increased compared to the RTO treatment. Our result demonstrated that soil chitinolytic community composition was obviously varied with tillage practices (*p* = 0.01), based on weighted UniFrac distance matrices (Fig. 6D). Meanwhile, our result further proved that RTO treatment OTUs showed obvious differences from compositions under CT (*p* = 0.028) and RT (*p* = 0.035) treatments.

**Fig. 8:**
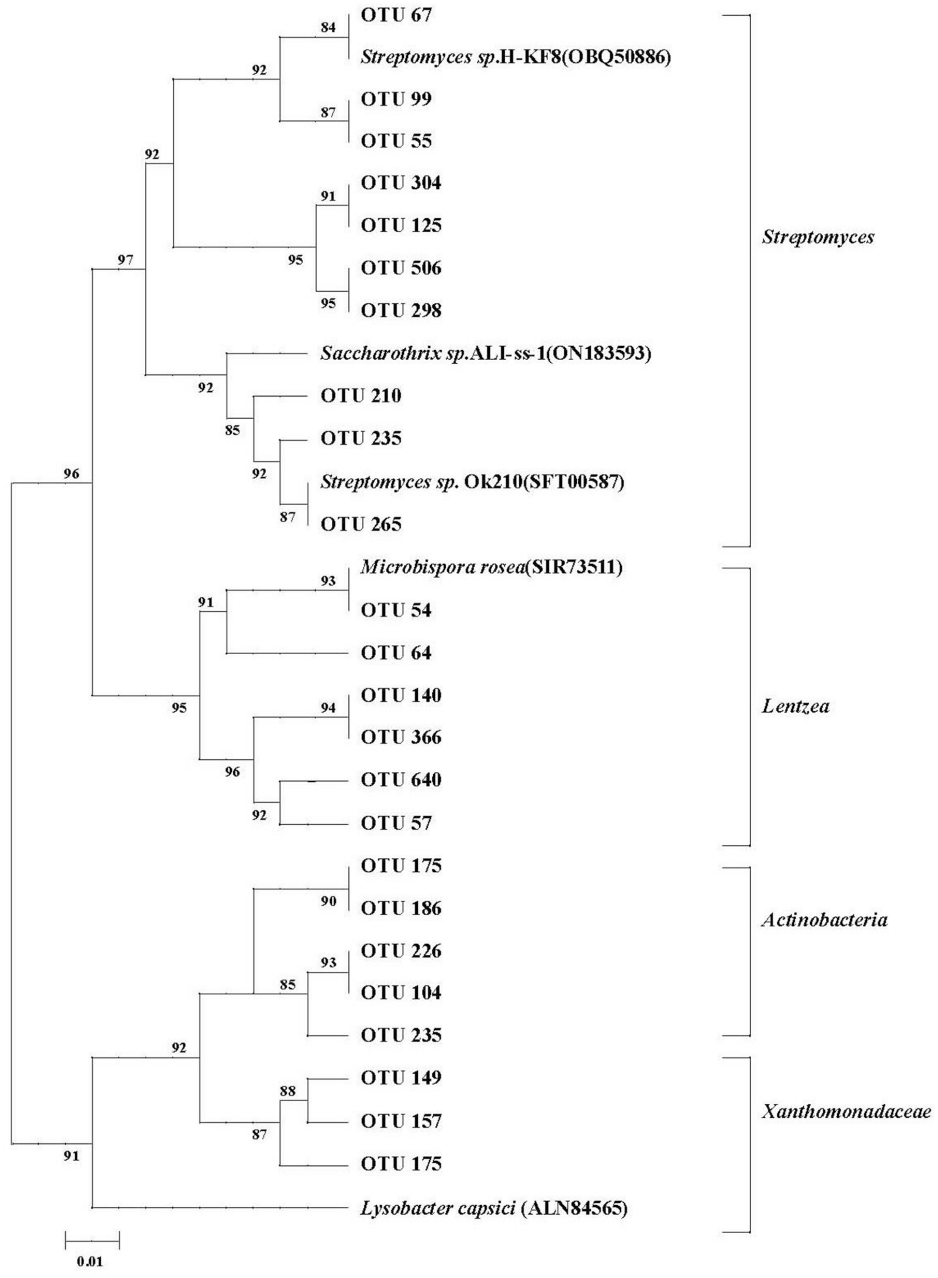
Phylogenetic tree of the top 510 most abundant partial *chiA* OTUs. Different branches indicated different taxa of *chiA*. OTUs: Operational taxonomic units.

This result indicated that *chiA* OTUs were mainly distributed among *Actinobacteria* with family members coming from *Actinoplanes* (*e.g.*, OTU 226), *Lentzea* (*e.g.*, OTUs 64, 140) and *Streptomyces* (*e.g.*, OTUs 125, 235). The most abundant OTUs were 149 and 175, which belonged to *Xanthomonadaceae*. In the top 510 *chiA* OTUs, abundances obviously varied under different tillage conditions (Fig. 8).

## Discussion

There is a positive correlation between soil nitrogen (N) and soil microorganism characteristics [30]. In this study, our results showed that rhizosphere soil N transformation rates (GMR, GACR, GNR, GNCR and NMR) in double-cropping rice field with RT, CT, and NT treatments were significantly improved. The reason may be attributed to a lower level of soil organic matter (SOM) and soil quality under no-crop-straw incorporation conditions. Secondly, soil chemical properties (*e.g.*, soil N, SOM contents) were enhanced under tillage and crop straw input conditions [19], which suggests that SOM provides more soil nutrients for rice. Furthermore, our results proved that soil N mineralization rates with RT and CT treatments were higher than that of NT treatment, indicating that soil chemical properties (soil N and SOM contents) were deficient with no-tillage and crop straw input treatments [4, 20]. Meanwhile, our results proved that soil N transformation rates in rice field with CT treatments were higher than that of RT treatment, which suggested the existence of higher levels of soil soluble N, and a larger soil active N pool in rice fields under conventional tillage condition, in addition to improved quality of SOM with crop straw input practice. Therefore, soil physicochemical properties and ecological environment were also improved with CT practices. Our results agreed with the previous studies [13], whose results proved that the degree of soil N mineralization in rice field with RT and CT practices was obviously improved, compared to NT practices, as they had similar C:N ratio and N content among these crop straw input treatments. Meanwhile, our results demonstrated that there was a significant, positive correlation between soil enzyme activities (*e.g.*, soil urease, protease, *β*-glucosaminidase and arginase) and soil N mineralization rate (*p* < 0.05), which were also in agreement with the previous study [17]. Therefore, differences in soil enzyme activities among these tillage management practices were the main factor influencing rhizosphere soil N mineralization in rice field.

In this study, our results proved that soil enzyme activities in rice field with RT and CT practices were significantly improved, which suggested that soil microbial growth and extracellular enzyme-organo complex activities were also improved [31]. Secondly, soil physicochemical properties and the ecological environment were also improved, which provided more nutrients for soil enzyme activities. Meanwhile, our results demonstrated also that N mineralization functional gene abundances were obviously changed with tillage treatments, and there was a positive correlation between soil N mineralization functional gene abundances and soil enzyme activities. The reason may be attributed to the fact that the functional genes included all members of the soil microbial community responsible for the related enzyme functions. The proteolytic gene primers for soil microbiota were developed and identified, and included neutral metallopeptidase (*npr*) and serine peptidase (*sub*) [32]. Meanwhile, these primer pairs used in the present study were applied to cover fungal communities. On the other hand, it was a feasible strategy to evaluate the relationship between abundances of N mineralization functional genes and their enzyme functions by using proteomic or RNA-based techniques [31]. Third, soil extracellular enzyme activities were controlled by genes encoding corresponding enzymes, and their stability and degradation were usually regulated by soil physicochemical properties.

Our results demonstrated that the structure of rhizosphere soil bacterial community in rice field was significantly changed with CT and RT practices. The richness and diversity of soil bacterial community in rice field were also strongly improved with CT, RT, and NT management, and these findings are in agreement with those of previous studies [2, 6]. The reason may be that native soil bacteria growth was stimulated under higher contents of soil available nutrients and diverse organic carbon fraction conditions [19]. In our study, more than 50% of OTUs from the no-crop-straw input practice were recovered in tillage and crop residue input-treated soils. Furthermore, the direct introduction of external source species to the rice field probably led to an increase of soil microbial community diversity, although the microbes were derived from crop straw input under short-term experiment conditions [13]. Meanwhile, our result proved that soil bacterial community diversity in rice field with RT and CT treatments was significantly higher than that of NT treatment, which was consistent with previous studies [14]. The reason was due to repeatedly planted rice and returned straw as an N source based on a no-tillage field experiment. Repeated cultivation of rice and returned crop straw into the paddy field may homogenize soil microbial communities, favoring soil microbes that are resistant to change due to no-tillage conditions [14].

In this study, our result proved that soil ureolytic community compositions in rice field with NT practice were significantly changed. Meanwhile, the soil ureolytic microbial community in rice field with RT and CT practices was also obviously altered. Our previous findings proved that there were obvious differences in SOC content between RT, CT, and NT management [20]. In this study, we found that more than 50% of OTUs from crop straw input were present in RT and CT treatments soils. These microbes inhabit organic manure and may play a vital role in regulating soil ureolytic community compositions under RT and CT treatments. Meanwhile, our results showed that abundances of the top 15 OTUs were obviously altered with tillage and crop straw input treatments. Furthermore, five of those affected OTUs with RT and CT treatments were improved, but abundances of those affected OTUs with RTO treatment were reduced. Therefore, our result showed that soil ureolytic microbes were inhibited under short-term, no-crop-straw input conditions. Meanwhile, our result proved that there was an obvious (*p* < 0.05) positive relationship between soil N mineralization rates and ureolytic community composition, which was consistent with previous results [8], which also showed that soil ureolytic community composition were closely related with soil N mineralization rates (GMR, GACR, GNR, GNCR and NMR). The reason was attributed to the soil community composition being controlled by genes encoding corresponding soil enzymes, and soil N mineralization rates regulated by soil physicochemical properties under different tillage conditions.

We also found that the soil ureolytic community mainly belonged to *Proteobacteria*, which was in accordance with the previous result [33], which found that soil bacterial urease usually exists in *Proteobacteria*. Meanwhile, our result showed that many *ureC* OTUs were not identified in the current reference, which suggests a need for more information on formerly uncultivated ureolytic organisms based on primer-based amplicon sequencing. But soil *ureC* sequences mainly belonged to *Thaumarchaeota* according to amplicon sequencing, although the abundances of *Thaumarchaeota* were small. Our previous result indicated that soil AOA was abundant in paddy field [34], which often contains *ureC*. However, previous results also demonstrated that potential producers of urease were mainly *Nitrospira*, since the top *ureC* OTUs were from *Nitrospira*, and some of them perfectly matched with the ammonia-oxidizing bacterium *Nitrospira inopinata* [35].

Previous findings showed that the soil bacterial chitinolytic community was significantly changed by the tillage management undertaken [36]. It is generally believed that soil chitinolytic community changes according to tillage and crop straw input management since crop straw contains multiple organic N polymers. This result demonstrated that abundances of several top *chiA* OTUs with CT and RT practices were obviously increased (Fig. 8). Furthermore, RT-, CT-, and NT-treated soils (crop straw input) were obviously different from the RTO-treated soil (without crop straw input). The reason was mainly due to a high variability in the composition of the chitinolytic community in RT and CT treatments. Meanwhile, our result showed lower levels of *chiA* OTU alpha diversity with NT treatment soil, indicating that the indigenous chitinolytic community was more competitive than chitinolytic microorganisms in no-tillage soil and exhibited weak survival in paddy soil. Several top *chiA* OTUs were obviously promoted with crop straw input-based treatments (OTU 149 and OTU 366), which indicated that the soil chitinolytic community in rice field may be stimulated under tillage conditions. Therefore, it is a positive strategy to promote rhizosphere soil N mineralization, soil ureolytic and chitinolytic bacterial community diversity, and soil enzymes activities under double-cropping rice system with rotary, conventional tillage, and crop straw input practices.

## Figures and Tables

**Table 1 T1:** Impacts of tillage treatments on rhizosphere soil N transformation rates in double-cropping rice field.

Items	Treatments			
	CT	RT	NT	RTO
GMR (mg N kg^-1^ d^-1^)	1.81 ± 0.05a	1.67 ± 0.05b	1.55 ± 0.05b	1.32 ± 0.03c
GACR (mg N kg^-1^ d^-1^)	2.62 ± 0.07a	2.44 ± 0.07b	2.23 ± 0.06c	2.02 ± 0.06d
GNR (mg N kg^-1^ d^-1^)	0.82 ± 0.02a	0.63 ± 0.02b	0.43 ± 0.02c	0.24 ± 0.01d
GNCR (mg N kg^-1^ d^-1^)	0.57 ± 0.02a	0.44 ± 0.01b	0.32 ± 0.01c	0.21 ± 0.01d
NMR (mg N kg^-1^ d^-1^)	0.58 ± 0.02a	0.47 ± 0.02b	0.30 ± 0.01c	0.12 ± 0.01d
NNR (mg N kg^-1^ d^-1^)	0.62 ± 0.02a	0.52 ± 0.02b	0.35 ± 0.01c	0.15 ± 0.01d
RR (mg N kg^-1^ d^-1^)	7.17 ± 0.18a	7.02 ± 0.20a	6.34 ± 0.21b	4.17 ± 0.12c

RT: rotary tillage with crop straw input; CT: conventional tillage with crop straw input; NT: no-tillage with crop straw returning;

RTO: rotary tillage with all crop straw removed as a control.

GACR: gross ammonium consumption rate; GMR: gross N mineralization rate; GNCR: gross nitrate consumption rate; GNR: gross nitrification rate; NNR: net nitrification rate; NMR: net mineralization rate; RR: respiration rate.

Values were presented as mean ± SE.

Different lowercase letters among different tillage treatments indicated significant difference at 0.05 levels.

The same as below.

**Table 2:** Impacts of tillage treatments on rhizosphere soil abundances of *sub*, *npr*, *chiA* and *ureC* in doublecropping rice field.

Gene abundances	Treatments			
	CT	RT	NT	RTO
Soil *sub* (copies × 10^7^ cells g^-1^)	3.85 ± 0.12a	3.71 ± 0.11a	2.86 ± 0.10b	2.41 ± 0.07c
Soil *npr* (copies × 10^5^ cells g^-1^)	2.91 ± 0.09a	2.78 ± 0.08a	2.46 ± 0.07b	2.23 ± 0.06b
Soil *chiA* (copies × 10^8^ cells g^-1^)	3.05 ± 0.14a	2.95 ± 0.11a	2.58 ± 0.09b	2.24 ± 0.07c
Soil *ureC* (copies × 10^7^ cells g^-1^)	10.25 ± 0.32a	9.68 ± 0.26b	8.93 ± 0.27b	7.47 ± 0.23c
